# Ethical Challenges of Using Virtual Environments in the Assessment and Treatment of Psychopathological Disorders

**DOI:** 10.3390/jcm10030378

**Published:** 2021-01-20

**Authors:** Thomas D. Parsons

**Affiliations:** 1 iCenter for Affective Technologies (iCAN), University of North Texas, Denton, TX 76207, USA; thomas.parsons@unt.edu; 2 Computational Neuropsychology and Simulation (CNS), University of North Texas, Denton, TX 76207, USA; 3 College of Information, University of North Texas, Denton, TX 76207, USA

**Keywords:** ethics, virtual reality, technologies of the extended mind, cyberpsychology, digital psychology, algorithmic devices

## Abstract

Clinicians are increasingly interested in the potential of virtual environments for research and praxes. Virtual environments include both immersive and non-immersive simulations of everyday activities. Moreover, algorithmic devices and adaptive virtual environments allow clinicians a medium for personalizing technologies to their patients. There is also increasing recognition of social virtual environments that connect virtual environments to social networks. Although there has been a great deal of deliberation on these novel technologies for assessment and treatment, less discourse has occurred around the ethical challenges that may ensue when these technologies are applied clinically. In this paper, some of the ethical issues involved in the clinical use of novel technologies are discussed.

## 1. Introduction

Virtual environments (VEs) are increasingly available for assessment and treatment of psychopathological disorders. In a virtual environment, patients can take part in a digital simulation of daily activities. There are varying degrees of exposure that a therapist can use when working with patients. For example, some clinical applications are non-immersive presentations of scenarios on a computer screen. Immersive virtual reality simulations involve head-mounted displays (HMDs) with head-tracking of the patient’s head position and movement. There are also highly immersive room-sized CAVEs (C-automatic virtual environments).

### 1.1. Virtual Environment of Things

While much of the work in VEs has focused on presentations of simulations to individual patients, the rise of social VR and the Internet of Things (IoT) connects everyday objects (including virtual simulations) to the Internet and enables data transfer from network-connected devices to remote locations. Furthermore, it allows clinicians to remotely administer assessments and treatments. Again, the majority of clinicians using virtual environments (for assessment and intervention) administer them in their clinics using a head-mounted display (HMD) and log the data locally. However, the rise of telepsychology and social virtual reality calls for increased attention to a Virtual Environment of Things (VEoT) that integrates real-world smart technologies with virtual objects and avatars in interactive and adaptive virtual environments [1,2,3].

### 1.2. Virtual Reality Exposure Therapy

Virtual reality exposure therapy (VRET) is one of the most common clinical applications of virtual reality. Clinicians use VRET to expose patients to a computer-generated virtual environments with clinically relevant stimuli (e.g., threating stimuli presented to patients with specific phobias) in a safe and controlled environment. Systematic reviews of VRET studies have revealed that clinically relevant digital simulations can enhance the effects of treatment sessions [4,5]. Likewise, there is growing emphasis in online administration of VRET. For example, Levy and colleagues [6] used an online VE scenario to treat acrophobia. They found the remote sessions to be well accepted and no major technical problems were reported. Moreover, psychological measures (e.g., anxiety, presence, therapeutic alliance) were comparable across the two conditions. In addition to phobias, mobile VRET is being applied to patients experiencing pain [7]. Studies have revealed that portable VE systems can be used to reduce chronic pain (reduced autonomic arousal and self-reported pain levels [8]. Other studies have automated VRET sessions and compared their efficacy to therapist-led VRET. For example, Lindner and colleagues [9] made this comparison and found that both therapist-led and self-led VRET sessions using mobile devices groups reported significant reductions in public speaking anxiety. Similarly, Freeman and colleagues [10] examined the efficacy of automated VRET sessions for acrophobia over a period of four weeks. They found that participants taking part in these automated sessions reported significantly reduced fear of heights.

### 1.3. Need for Training in Ethical Use of Technologies

As clinicians are adopting technologies for delivery of services and practice management [11,12], there are related needs for discussion of ethical challenges that may hinder the process, efficacy, and even security of assessment and treatment. These challenges may go beyond the limited training received by the majority of clinicians. As such, they may be unprepared for ethical challenges (e.g., privacy, electronic security, legal implications) involved in using virtual environments. This lack of preparedness is compounded when considering telepsychology applications and VEoT. Likewise, clinicians interested in VEs will likely want recommendations beyond those provided in professional ethical guidelines for telepsychology from the American Psychological Association [13] and International Society for Mental Health Online [14]. In addition to guidelines, clinicians interested in VEs may benefit from continuing education for the consistent upgrades to available technologies (e.g., VRET; VEoT; algorithmic devices).

Practice guidelines developed for both face-to-face therapy and teletherapy approaches may have limited generalizability to clinical practices using VEs. Herein, potential ethical concerns are considered for clinicians using virtual environments and their interactions with clients. Included will be considerations about whether a client’s disposition and/or situation call for virtual reality-based assessment and/or therapeutic intervention. The discussion starts with considerations of legal and ethical concerns surrounding confidentiality (e.g., privacy) and electronic security. Emphasis is placed on the application of ethical codes and guidelines. Following this discussion, special considerations for using adaptive virtual environments that act as algorithmic devices extending cognition will be considered.

## 2. Ethical Issues in the Clinical Use of Virtual Environment Technologies

General discussions of the appropriate use of virtual environments for non-clinical applications are found in legal codes [15], ethical guidelines [16,17], and ethical codes of conduct [18,19]. More specific ethical considerations include privacy [20,21] and representation [22]. These discussions can also be found in discipline specific areas such as virtual reality games [23], virtual human avatars [24], and cyberlearning [25,26,27,28]. While there have been discussions of clinical applications of virtual reality [29,30,31,32,33] and the convergence of VEs with social networks [34], there is further need for emphasis on ethical challenges for the clinical use of VEs for both assessment and treatment of psychological disorders in the IoT era [35].

### 2.1. Legal Issues Related to Technology Use in Clinical Praxis

It is important to note from the outset that regulatory structures have been developed for clinicians interested in using technologies in research and practice. In addition to those set forth by the American Psychological Association [13], there are acts like the Health Information Portability and Accountability Act (HIPAA) [36] that offer regulatory frameworks that can guide clinicians in the use of appropriate protections for safeguarding a patient’s digital medical privacy. Clinicians considering the use of VEs for assessments and interventions must follow HIPPA guidelines in determining potential risks associated with various VE platforms. In addition to presenting complex and interactive stimuli, VE platforms can log patient responses into databases that can be uploaded to third party cloud storage services. While not all clinicians will be interested in this ability, those who are will want to consult the Health Information Technology for Economic and Clinical Health Act (HITECH Act) [37] that focuses on privacy and security risks involved in electronic transmission of health information. A related regulatory structure is the Family Educational Rights and Privacy Act (FERPA) of 1974 [38], which regulates access to information (e.g., educational records) by public entities (e.g., prospective employers, public organizations, and foreign governments). This regulatory structure is especially important for clinicians working with students. Finally, clinicians need to learn and apply the American Psychological Association’s guidelines, codes, and licensure jurisdiction of use. This may involve attendance at technology-oriented workshops and continuing education programs that focus on legal considerations, as well as consulting with attorneys who specialize in healthcare policy and privacy.

### 2.2. Ethical Principles for Clinicians Using Technologies

The majority of clinicians will have received ethical training in a course or two that featured practice guidelines [13,14,39,40,41] and case examples. Part of this training will likely include the Nuremburg Code [42], the World Medical Association’s Declaration of Helsinki [43], and the Belmont Report [44]. Clinicians are commonly informed of three principles undergirding several contemporary ethical guidelines: respect for persons, beneficence, and justice. Relatedly, there is often presentation of Beauchamp and Childress’s [45] four ethical principles: (1) Autonomy or patient right to choose or refuse treatment (informed consent); beneficence (clinician acts in the best interest of the patient; nonmaleficence (clinician aims to do no harm (minimize risks); and justice (clinician fairly distributes benefits and burdens).

From these principles, the American Psychological Association’s Ethical Principles of Psychologists and Code of Conduct [46] offers five principles: (1) Beneficence and non-maleficence (i.e., minimize costs and maximize benefits; protection from harm); (2) fidelity and responsibility (professionalism; societal obligation); (3) integrity; (4) justice; and (5) respect for patient’s rights and dignity (e.g., privacy and confidentiality). Each of these sets of guidelines offers standards for the ethical use of technologies in clinical care. Moreover, these guidelines emphasize the need for technical aspects of the technology that must be learned and implemented to safeguard patients (e.g., privacy settings and encryption).

## 3. Risks and Recommendations for the Clinical Use of Virtual Environments

The use of VE platforms requires the clinician to limit potential adverse side effects that can limit the efficacy of virtual environments for certain cohorts (e.g., clinical populations; younger/older age participants). Early ethical considerations by Behr and colleagues [16] suggested four potential risks: (a) Simulator (i.e., motion) sickness; (b) information overload; (c) experience intensification (VE intensifies arousal that may strain frustration tolerance), and (d) dysfunctional re-entry into the real world following VE exposure. In the years since Behr and colleagues proffered these risks, VEs have become increasingly realistic and further risks have immerged with this realism [19,47]. As such, there is growing need for discussion of additional clinical risks, informed consent, the convergence of VEs with the Internet, and algorithmic devices (e.g., smart technologies).

### 3.1. Simulator Sickness

Simulator sickness (also known as cybersickness) is similar to the symptoms found in motion sickness. Some patients may have greater sensitivity to being immersed in a virtual environment. These individuals experience an unpleasant side effect that can be manifest as motion sickness with fatigue, headache, eye strain, and/or nausea [48,49]. Simulator sickness symptoms can occur alone or together during and/or post-immersion in a virtual environment [50,51,52]. While the actual neurophysiological underpinnings of VR-based simulator sickness are not well established, there is some evidence suggesting that sensory mismatch and postural instability are potential causes [53]. When working with clinical populations, it is important to note that some patients with underlying neurological conditions may be have increased susceptibility to simulator sickness (e.g., multiple sclerosis) [54]. Various approaches to decreasing simulator sickness have investigated: situating static rest frames on the virtual scenery [55], decreasing field of view [56], and clouding rotational movement [57]. A potential area of promise for alleviating simulator sickness is found in adaptive algorithms that could reduce cybersickness via learning algorithms for real-time adaptation of simulations relative to the patient’s experience [58]. When coupled with (neuro)physiological parameters (e.g., eye-movement, pupillometry, heart rate, electroencephalography), closed-loop VE platforms (i.e., VE simulation is adaptively modulated relative to the patient’s behavioral responses and neurophysiology in real time) can be developed for real-time detection of simulator sickness, as well as adaptation of the virtual environment to lessons symptom severity [59]. In addition to alleviating simulator sickness, these closed-loop systems could offer improved autonomy (and agency) for patients with neurological or psychiatric disorders that limit everyday activities (e.g., dyskinesia, debilitating anxiety).

### 3.2. High Fidelity Stimulus Presentations, Experience Intensification, and Information Overload

Even back in 2005, Behr and colleagues [16] expressed concerns about the vast amounts of visual, aural, tactile, and/or even olfactory information presented in virtual environments. Recently, 15 years later, Slater and colleagues [47] point to the “superrealism” of today’s VE platforms. These VEs have greatly enhanced the visual realism via stereoscopic vision, head tracking, and eye tracking. Moreover, there is increasing fidelity in immersive sound rendering and haptic rendering, as well as smell machines (olfactory). Ramirez and colleagues [60,61,62] contend that if the superrealism of a VE simulation effectively recreates real-world scenarios, then the VE protocol should be subject to the same human subjects’ concerns found in real life protocols. While this remains a philosophical (and technological) discussion, it does raise concerns for clinicians working with vulnerable populations. As such, clinicians need to remain apprised of these technological advances, the potential overload that may impact their patients, and closely watch for visual and behavioral responses to superrealism. The consequences of sensory and informational overload can be ethically problematic because they may impact the patient’s autonomy/self-determination and the principle of nonmaleficence.

### 3.3. Depersonalization, Derealization, and Dysfunctional Re-Entry into the Real

Concerns have been raised related to the potential of VEs for depersonalization and derealization because virtual environments are designed to manipulate the cognitive and affective mechanisms involved in generating virtual experiences that replicate real experiences. The dissociation that occurs is similar to that experienced by persons with clinical compromise. Even if a VE user does not embrace the virtual environment as something more than a “virtual reality”, immersion in high-fidelity simulations can engender illusory experiences that feel “as if” the virtual reality is real. For example, when participants are immersed in a virtual environment that simulates standing at the edge of a deep pit, an elevated ledge, and walking a plank, their autonomic responses (heart rate and skin conductance) indicate significant stress elevations [63,64,65,66,67].

From an ethical perspective, these simulations offer the potential for both positive and negative outcomes. On the one hand, clinicians have effectively treated various phobias with VRET in general [68,69,70,71,72,73] and with acrophobia (fear of heights) [74,75] in particular. In such situations, a clinicians may reason that they are acting in the best interest (beneficence) of their clients when exposing them (gradually) to fearful stimuli in a controlled virtual environment. That said, there are situations where exposing a client to a virtual environment can be overwhelming. As mentioned earlier, there are strong psychophysiological reactions that occur when persons are immersed in simulations with fear-inducing stimuli. The therapist needs to be very clear in both their informed consent before therapy, and monitoring of patient responses throughout exposure. Moreover, long-term exposure and immersion may negatively impact neural mechanisms and autonomic responding as persons with a proclivity toward dissociation experience derealization. Some argue that the experience of being immersed in virtual environments is similar to symptoms found in dissociative disorders (depersonalization and derealization) [17,76,77]. As a result, clinicians must consider the risks of immersing some clients into virtual environments that may interfere with autonomy (agency and responsibility) needed for judgement and decision making.

### 3.4. Virtual Environments with Vulnerable Populations

The use of virtual environments for VRET and VEoT (telepsychology, eTherapy) interventions with vulnerable populations (children, older-aged, at-risk patients) necessitates discussion of cohort-specific ethical concerns. Moreover, there are situations in which clinicians will use virtual embodiment for treating clinical populations who present with a distortion in their internal body representation. As Madary and Metzinger [19] point out, it is important to consider the ethical implications of virtual embodiment because it can lead to cognitive, affective, and behavioral changes. Moreover, the clinician’s use of VR to manipulate patients’ perceptions of their bodies may have unintended results, or even pain (e.g., VR-induced phantom limb pain) [47]. Hence, clinicians must practice even greater diligence when considering the ethical risks of using technologies with patients that may have difficulties in understanding consent and the various issues involved in being treated with simulations. Two significant matters for ethical consideration when working with special populations and virtual environments are: informed consent and protection. There are various opinions about whether it is enough to obtain consent from parental/legal guardian/caregiver, or should the participants always also be asked for their consent. Some contend that vulnerable patients (e.g., children) should be invited to offer consent [78]. According to the APA Code of Ethics, even when a patient is legally incapable of giving informed consent (e.g., children, older adults) clinicians should still request their assent [79].

An important note for clinicians working with vulnerable populations is that these patients have the same rights of withdrawal from participation that would be afforded to non-vulnerable clients. Moreover, these patients should experience the same data protections, confidentiality, and privacy that others experience. Clinicians will need to manage disclosure of adverse impacts as and when they arise. The consenting process ought to contain clear and precise descriptions of what the treatment entails, the potential benefits and side effects, as well as alternative therapeutic options. Moreover, the clinician should inform the patient that while anxiety may initially increase at the beginning of the intervention, cumulative exposure is aimed at enhancing their tolerance and helping them better control their anxiety (improved autonomy).

### 3.5. Therapeutic Misconceptions

A related issue for protecting vulnerable patients is balancing the costs and benefits for special populations. Here there is the issue of therapeutic misconceptions that patients may have about what virtual reality interventions can actually offer [80,81,82]. While VRET has been shown to be efficacious for various phobias in general, more research is needed to see how well VRET treats patients in specific cohorts. The question for clinicians is whether a patient from a vulnerable cohort actually need expensive VRET (i.e., costly hardware/software platforms) and potentially risky interventions when much less expensive (with less ethical risks) face-to-face therapies are equally efficacious. While results reveal clinical improvements in anxiety symptoms after VRET in adults, VRET efficacy for children and adolescents with anxiety disorders is not well established. More research (especially randomized clinical trials) is needed with younger cohorts [83]. Another example can be found in veterans experiencing trauma symptoms. A clinician may have a patient who experienced combat stress symptoms (i.e., post-traumatic stress disorder) and is seeking help with affective dysregulation. The military service member may have a therapeutic misconception that the uniqueness of VR will have greater impact on their trauma symptoms than traditional face-to-face interventions. A concern here (as with any novel intervention with limited validation) is that the research may not support the potential costs. Evidence calling into question the benefits of VRET for veterans can be seen in findings from a randomized clinical trial comparing the efficacy of VRET with traditional prolonged exposure therapy (i.e., talk therapy) for treating posttraumatic stress disorder. The trial included a large cohort (N = 162) of active duty soldiers with combat-related trauma [84]. Findings revealed that talk therapy (using prolonged exposure) was superior to the more expensive virtual reality exposure. The superiority of talk therapy was evident in greater alleviation of symptoms at three- and six-month follow-ups. Hence, clinicians using virtual reality must way the costs and benefits of applying VR to their interventions with certain populations. Furthermore, there is a need for much more research into the use of VR for vulnerable populations.

## 4. Virtual Environments for Assessment

In addition to applications like VRET, clinicians (e.g., clinical neuropsychologists) are increasingly developing and validating virtual environments for neurocognitive and affective assessment [85]. Virtual environment-based neuropsychological assessments offer high-dimensional tools for assessing both cognitive constructs and real-world functional capacities. These virtual environment platforms offer potential for improving the ecological validity of neuropsychological assessments [86,87] through accurate and controlled presentation of dynamic/three-dimensional perceptual stimuli. Moreover, using VEs clinicians can balance ecologically validity and experimental control of specific ecologically valid tasks. High dimensional VR platforms offer immersive simulations with enhanced stimulus presentations that reproduce the distractions, stressors, and/or demands found in everyday activities.

An important ethical consideration for clinicians interested in virtual environment based neuropsychological assessments is the lack of adequately norms and related dearth of psychometric validation. While there are increasing efforts aimed at psychometric validation of virtual reality based neuropsychological assessments [88,89,90], clinicians must use their ethical judgements to balance the added understanding of the patient’s performance of activities of daily living (from the VE) with the lack of adequate norms. Before virtual environments can be widely adopted, there is need for focused guidelines on the development (design issues and manuals), norming, psychometric validation, and administration of these VE platforms [91,92,93]. From an ethical perspective, much more research is needed before clinicians can rely on virtual environments for their assessments. Ethically appropriate use will be aided by psychometric validation via large-scale randomized clinical trials. For now, patients are best served when clinicians use traditional paper-and-pencil neuropsychological batteries that are well validated (psychometrically) and can adequately measure cognitive constructs. Virtual environments can be added to these traditional batteries for both validation (of the VEs) and treatment recommendations (from patient performance in everyday activities simulated by the VE).

Clinicians may encounter ethical challenges when conducting virtual reality-based assessments and interventions. An essential tension exists between the ethical principles of beneficence (maximizing patient benefit) and nonmaleficence (avoidance of harm). Clinicians face this dilemma when choosing to immerse a patient into a virtual environment for an extended period (e.g., assessment) and/or over a series of treatments. On the one hand, a VR-trained clinician may be inclined to administer a well validated (manualized treatment validated via randomized clinical trials) VRET to a patient in an effort to maximize the patient’s well-being (beneficence). On the other hand, immersing a patient into a virtual environment can result in adverse reactions (e.g., simulator sickness, dissociation) that actually counteract therapeutic efficacy (nonmaleficence). Clinicians considering the use of VR for assessment and/or treatment should meet with the patient and inform them of the potential benefits and risks. Included in this discussion should be a consideration of the nature and severity of the patient’s distress and the patient’s comfort with technologies. Moreover, the therapist can expose the patient to trial runs of the virtual environments (equipment, controllers, virtual scenario) prior to actual assessment or therapy. Then, clinicians can converse with patients about the patient’s experience. This approach allows the clinician to work with the patient to balance patient benefits while minimizing harm. It is important to note that just because a patient is comfortable with technology, the nature and the severity of the patient’s presentation may still contraindicate the use of a virtual environment. For example, a patient presenting with severe personality disorder, psychotic disorder, suicidality, and/or homicidality may not be a good candidate for virtual environments.

## 5. Telepsychology and Virtual Environment of Things: Privacy and Confidentiality

Along with the Internet age comes a growing rise in the IoT. Clinicians need to be aware of the increasing reality that their online activities are consistently monitored, logged, and shared. Virtual environments already gather a good deal more personal information (when compared to traditional face-to-face talk therapy) about the patient’s (and/or research participant’s) eye-movements, behavioral response patterns, and motor responses that make up a patient’s “kinematic fingerprint” [17]. The addition of IoT, algorithmic devices, and social VR leads to additional ethical concerns related to the logging and sharing of the patient’s habits, interests, and tendencies. The potential for logging and sharing personal data may threaten personal privacy. Concerns related to ethical risks are heightened by the ongoing convergence of virtual reality and social networking (VRSN) [34]. O’Brolcháin and colleagues [34] have discussed the ethical considerations involved in VRSN and identified three general areas with threats to privacy: (1) Informational privacy (third party access to patient’s digital footprint—personal information, psychological features, financial, medical and educational records), (2) physical privacy (third party sensory access to a patient’s body and activities; associated ethical issues are modesty, autonomy, bodily integrity), and (3) associational privacy (difficulty in controlling who one is interacting with in VEs).

The progression of VRSN, VEoT, and wearable sensors (e.g., eye-tracking; psychophysiological metrics) makes privacy an increasing concern. There are important ethical concerns related to the privacy and confidentiality of patients involved in telepsychology (e.g., eTherapy; online research) [94,95]. Vulnerabilities in patient information (electronic communication records, electronic patient data transfer; informational notices, and patient waivers) abound in VRSN, VEoT, and telepsychology. Professional organizations often assign blame to the service providers [13,79] and clinicians need to use HIPPA compliant platforms. Clinicians are also held responsible for informing patients of the limitations of technologies used and related limits to patient confidentiality when patient data is transmitted electronically. To secure electronic data transmissions from third party interception without patient consent requires that the clinician encrypt data transmission [96]. Moreover, clinicians should make sure that devices are password-protected to safeguard the patient’s meta-data (e.g., email addresses; phone numbers) and confidential information (voicemails and other communications) [96]. Parsons, McMahan, and Kane [91] offer practice parameters to maintain confidentiality. They also discuss software and hardware configurations that may impact telepsychological practices. Of note is their delineation of optimal procedures for limiting errors in the design, development, and administration. Clinicians need to use platforms designed by developers who made available bench test results for their software’s performance on various devices and minimum specifications (documented in manuals).

## 6. Informed Consent

Several codes of ethics and ethical guidelines have been developed and professional societies have established specialty-oriented policies and guidelines. Much of this work has emphasized protections against research-related harm, violations to autonomy, and risks. An important component is informed consent that must be obtained from research participants and/or patients. The consenting process is to be completed as soon as possible [79]. The informed consent should make every effort to use language that patients (and research participants) can understand. According to the American Psychological Association’s ethics code [79] informed consent requires the clinician to inform patients (as well as research participants) about (1) the research purpose, anticipated duration and procedures; (2) the patient’s (and/or research participant’s) right to decline participation and/or withdraw from participation; (3) the foreseeable consequences of declining or withdrawing; (4) any foreseeable influencing factors such as potential risks, discomfort, or adverse effects; (5) any potential benefits; (6) confidentiality limits; (7) incentives; and (8) contact information for questions about the research and participants’ rights. Moreover, participants should be provided with opportunities for asking questions and receiving answers.

Informed consent is a vital component in virtual reality-based neuropsychological assessments and virtual reality exposure therapy. Consent ensures that the patient (and/or research participant) understands the purpose of the virtual environment protocol, the procedures, and an estimate of the duration of the virtual reality exposure. Part of informed consent involves the clinician’s informing the patient (and/or research participant) of the patient’s right to decline participation in the virtual environment and/or withdrawal from the virtual reality exposure. This will also include any potential foreseeable consequences that might occur should the patient choose to decline or withdraw. Informed consent can be a collaboration between the clinician and patient (and/or research participant) who collaborate to determine shared goals and discuss any prospective clinical and or research benefits [97]. Collaborative considerations during the consenting process can increase treatment effectiveness, enhance cooperation, and bolster trust.

## 7. Special Issues with Adaptive Virtual Environments and Algorithmic Devices

In the earlier sections, much of the discussion surrounded ethical concerns for clinicians using virtual environments with patients. For example, a therapist may use VRET with a patient who has a specific phobia (e.g., arachnophobia). The clinician can immerse the patient into the virtual environment, monitor the patient, and then make decisions about gradual changes to the patient’s exposure. This is a very controlled situation, in which the clinician learns the patient’s cognitions, proclivities, and responses and uses that information to update and adjust the virtual environment. With the advent of smart therapeutic technologies and algorithmic devices/platforms (e.g., adaptive virtual environments), this therapeutic control can be bypassed. While this marks an advance in terms of personalization, amount of information logged and the response time of the therapeutic platform, it also poses ethical concerns.

### 7.1. Extended Cognition

The addition of smart algorithms to VRSN, VEoT, and wearable sensors can extend users’ cognitive and affective processes beyond the wetware of their brains [26,27]. We are already seeing this with the smartphones and the IoT that enable us to translate, recall, analyze, and compute information. They also enable us to navigate our environments. Much of this knowledge is publicly available via the Internet. Smart technologies also gather personal information (e.g., contacts, emails, text messages, posts, calendar appointments) and log everyday activities (purchases, readings, film viewing, steps taken, calories, and so forth). These smart technologies learn from their users, can be programmed to make suggestions, and extend the user’s cognitive processes.

These smart technologies are our latest attempt to offload our cognitive tasks into the environment. Dennett [98] contends that our notable evolutionary achievement is less a feature of our large frontal lobes, and more tied to our ability to extend our cognitive processes into the environment with which we interact. Clark and Chalmers [99] consider “extended cognition” to include complex feedback and feedforward loops among brain, body, and the peripheral world. Smartphones and the IoT form extended cognitive systems that can perform cognitive processes that would otherwise be realized via internal brain-based processes. They employ a “parity principle” as follows:

If, as we confront some task, a part of the world functions as a process which, were it to go on in the head, we would have no hesitation in recognizing as part of the cognitive process, then that part of the world is (so we claim) part of the cognitive process ([99], p. 8).

Discussions of the extended cognition paradigm can be found in psychotherapy [100], dementia [99,101,102], psychopathology [103], and depression [104]. Furthermore, specific applications have been discussed for treatment of borderline personality disorder [105], neurodevelopmental disorders (attention deficit/hyperactivity disorder, autism) [103] social anxiety disorder [106], dispositional affective states [107], sexual dysfunction [108], and sex offenders [109,110].

### 7.2. Technologies of the Extended Mind

The idea of extended cognition can be applied to technologies that extend cognitive processes beyond the brain. Reiner and colleagues [111,112] have referred to this coupling of humans with algorithmic devices as technologies of the extended mind. While they are interested in technologically extended cognition, they contend that not every algorithmic function carried out by technologies (external to the brain) qualifies as a technology of the extended mind. Instead, a relatively seamless interaction is needed between the brain and algorithms such that a user perceives the algorithm as being an extension of the user’s mind [111,112]. Over time, the repeated and regular use of an algorithmic device can engender automated and algorithmic coupling of mind (brain processes for cognitive, affective, and social functioning) and technology (e.g., smart technologies). For example, a therapist performing remote telehealth interventions could suggest an adaptive VEoT to a patient interested in using it at home to alleviate agoraphobia. This patient becomes very anxious while shopping and tends to forget previously memorized shopping items. When the patient first begins utilizing the new algorithmic VEoT, the patient may be careful to check and double check suggestions made by a virtual assistant in the VE and may continue to self-remind that the simulation of a crowded store is not real. Following Reiner and colleagues [111,112], this does not represent a technology of the extended mind because the patient continually questions the trustworthiness of the VE and virtual assistant. After using the VEoT several times over a period of weeks, the directions from the VE and virtual assistant are so trusted that the patient begins to rely on them when navigating the virtual shopping environment. Here there appears to be a relatively seamless interaction between the patient’s brain and the algorithms (causing actions in the VE and virtual assistant) that extend the patient’s cognition.

### 7.3. Virtual Reality-Based Memory Palaces for Extended Cognition

There is a growing body of literature that considers virtual environments to be technologies of the extended mind [32,101,113,114,115,116,117]. One example can be seen in developments of virtual reality-based memory palaces that were developed from the method of loci technique that uses an imagined palace for retaining information. Persons who use this approach associate each item of information with a location along a route in the visualized space. When aiming to retrieve a piece of information, a person can mentally retrace their steps through the location and then envision the element allocated to that location. Virtual reality-based memory palaces have been developed for virtual environments [118,119,120,121,122,123,124,125]. Peeters and Segundo-Ortin [113] have conceptualized a virtual reality-based memory palace as a technology of the extended mind that turns toward an embodied and enactive approach.

Given the potential for VEs to extend cognition, it is important to consider the ethical implications that may occur as the technologies develop. Take the example given earlier of a patient using a virtual environment to shop for various items (shopping list is learned before being immersed in the environment). A therapist could introduce the idea of memory palaces to the patient along with various mindfulness techniques for relaxation. The patient with fear of crowded public spaces could be given a list of shopping items for a friend’s birthday party and told to stay within a certain budget. The therapist may use a VEoT application that simulates a large grocery store that was modeled after a local grocery store in real life (see for example, [126,127]). Once immersed in the virtual environment grocery store, the VEoT system and virtual assistant communicates the best routes to aisles with specific shopping items. After arriving at an aisle, the user can learn facts about each item (e.g., price; nutrition information; expiration date) from a virtual assistant. This virtual assistant is especially advantageous as it monitors the user’s psychophysiology as the user navigates the aisles. Some aisles are crowded with virtual humans and the VEoT can make suggestions of routes with less avatars. At first, the patient may be uncertain of the VEoT because the patient is not that familiar with the technology. As a result, the user remains alert to surroundings and stays away from crowded areas.

After a few weeks of using the VEoT, the patient begins to trust the virtual assistant and seldom resists following the virtual assistant’s guidance. While the VEoT is executing computations external to the patient’s brain, the virtual assistant in the VEoT is probably better understood as a cognitive aide than a technology extending cognition. This is due to the fact that the VEoT’s calculations and the patient’s use of them are not acting as an automated cognitive loop with the user’s cognitive processes. After using the VEoT over an extended period, the therapist works with the patient to monitor the patient’s affective arousal as the patient uses the memory palace technique to remember the location of items in the store. Seeing that the patient is not experiencing elevated arousal levels, the therapist suggests that the patient go to an actual grocery store and use a mobile app that includes the virtual assistant from the VEoT. The virtual assistant never failed the patient in its directions to aisles or its information (e.g., price; nutrition information; expiration date) about the items in each section. At the grocery store, the patient searches for items using the smartphone application’s search interface and when the route is presented on the smartphone screen, automatically follows it to the aisle and attends to the virtual assistant’s advice about the shopping items. The smartphone application is starting to operate as a technology of the extended mind as the user is coupled with the algorithmic device.

## 8. Ethical Considerations for Technologies Extending Cognition

Assuming that technology can extend our cognitive processes into the external world, should clinicians apply the same ethical considerations that govern everyday practice to interventions that include extended mind loops. A potential ethical consideration for the patient’s (mentioned earlier) use of VEoT, virtual assistant, and smartphone application is that after using the applications for a period, the patient had assimilated the technology’s algorithmic processes into the patient’s own cognitive processes while shopping. What would the ethical considerations be if the smartphone sends the patient prompts when the patient passes a sign advertising a special for hair coloring; and again sends a chime (i.e., alert notification) when the aisle with the special is just up ahead. Here the ethical concern is that the algorithms have learned the patient’s preferences and are attempting to influence the patient’s actions. Further, the smartphone algorithm may strengthen its suggestive power by “questioning” whether the patient would like to get the hair care products. The patient is on a budget and only has enough money for the birthday party items on the shopping list. This creates some anxiety in the patient given that the patient has concern about making a good impression at the party. The conflict involves considerations about whether or not to stay within budget and get the items on the list. Here, there is the ethical concern that the technology is influencing the patient to the point that the patient experiences discomfort and may alter plans to complete shopping. While it can be argued that this is a fairly inconsequential case of undue influence, it still represents an autonomy violation. The clinician should consider the possibility that the algorithm extending the patient’s mind was designed by a corporate entity that may receive compensation by hair care vendors at the grocery store for directing the patient to them. Such probable conflicts of interest should be considered carefully when assessing the algorithm’s capacity for violating a patient’s autonomy.

## 9. Conclusions

In this review, current regulatory guidelines (HIPPA; HITECH Act, 2009; and the FERPA (or Buckley Amendment) Act) were considered that can be used to guide clinicians as they consider the ethical implications of using virtual environments. Moreover, clinicians were advised to absorb and apply American Psychological Association guidelines, codes, and licensure jurisdiction of use. To stay current in the digital era, clinicians need to attend technology-oriented workshops and continuing education programs. At times, clinicians will also need to consult with subject matter experts and attorneys who specialize in healthcare policy and privacy. Moreover, clinicians need to be able communicate legal protections to (and for) patients.

This review also considered the American Psychological Association’s (APA) Ethical Principles of beneficence and non-maleficence; fidelity and responsibility; integrity; justice; and respect for patient’s rights and dignity. Clinicians can use these APA principles when considering the use of technologies with their patients. It was noted that APA guidelines call for clinicians to only practice within the bounds of their knowledge of the technical facets of the technologies so that they can safeguarding their patients (e.g., privacy settings and encryption). These issues are even more important when working with special clinical populations. Here, clinicians must be ethically vigilant when using technologies. It is important that clinicians make specific efforts to provide informed consent and protection. The application of technologies to therapy with patients must be ethical, sensitive, respectful, and protected. Furthermore, clinicians must be careful when considering novel technologies and question the extent to which the technologies have been psychometrically validated.

While much of this discussion can be applied to the growing research base (and practice) of virtual reality exposure therapy [4,5,128,129], the advent of algorithmic devices (e.g., smartphones), VEoT, and virtual humans, add new ethical issues given potential for extending the patient’s cognitive and affective processes beyond the wetware of their brains. The idea of “extended mind” characterizes human cognizing as comprising complex feedforward and feedback loops among brain, body, and the external world. If technology does extend patient’s cognitive and affective processes into the external world, clinicians consider the ethical implications. Clinicians deliberating about whether to suggest algorithmic therapy devices to their patients will want to make sure that the patient is well informed and that patient privacy and autonomy are maintained. Furthermore, it is important that a patient not become over reliant on technologies.

In summary, this paper considered a number of concerns applicable to clinical practice using virtual environments. These reflections can guide the clinician’s considerations of judiciously use of technologies in clinical research and practice. Additionally, appropriate use of technologies, relevant legal and ethical issues, and maintaining patient privacy were discussed. Prior to suggesting technologies to patients, clinicians must have comprehensive understanding of privacy standards, confidentiality, and security. Finally, when using technologies and algorithms that can extend the patients cognitive processes, clinicians must consider the ethical issues from a technologies of extended mind framework.
